# A Cross-Sectional Study on the Prevalence of Premature Canities Among University Students in the Eastern Province, Kingdom of Saudi Arabia

**DOI:** 10.3390/medicina62020268

**Published:** 2026-01-27

**Authors:** Heba Y. Alojail, Irshad Ahmad, Feroze Kaliyadan

**Affiliations:** 1 Department of Dermatology, King Faisal University, College of Medicine, Al-Ahsa 36362, Saudi Arabia; 2 Department of Bioengineering, King Fahd University of Petroleum and Minerals (KFUPM), Dhahran 31261, Saudi Arabia; 3 Interdisciplinary Research Center for Membranes and Water Security, King Fahd University of Petroleum and Minerals (KFUPM), Dhahran 31261, Saudi Arabia; 4 Department of Dermatology, Amrita Institute of Medical Sciences and Research Centre, Amrita Vishwa Vidyapeetham, Ponekkara, Kochi 682024, India; ferozkal@hotmail.com

**Keywords:** epidemiology, haircolor, medical students, premature graying of hair, canities

## Abstract

*Background*: Premature graying of the hair (PMGH), commonly referred to as canities, is a condition that has both genetic and environmental causes, all of which are not fully understood, and is typically accompanied by severe psychological distress. Studies are limited regarding PMGH, with no clear guidelines available. *Methods*: A cross-sectional study was conducted during the academic year 2023–2024 among medical students in a college in Saudi Arabia. *Results*: Out of 239 medical students surveyed (mean age of 22.9 ± 5.3 years; 54.4% female), the prevalence of premature graying of hair (PMGH) was 37.2%. PMGH was significantly associated with smoking (80% vs. 34.8%; *p* = 0.003), keto diet (72.7% vs. 35.5%; *p* = 0.013), hair coloring (51.2% vs. 34.3%; *p* = 0.042), and family history of PMGH (47.7% vs. 20%; *p* = 0.001). Although our study did not directly measure psychological stress, the findings suggest that stressful factors and lifestyle changes common among medical students may contribute to the development of premature graying of hair. *Conclusions*: This study demonstrates that early hair graying is caused by both genetic and modifiable factors, and its incidence and psychosocial effects might be lessened with increased awareness and early lifestyle changes.

## 1. Introduction

Graying of hair represents a part of normal aging [1]. Premature graying of hair (PMGH), or canities, is defined as the onset of graying and is variously labeled as onset before the age of 20 in Europeans, 25 in Asians, and 30 years in Africans [2]. This condition represents a concern from an aesthetic point of view and could be an indicator of underlying systemic disease as well [3,4]. Premature graying is multifactorial in origin and is thought to involve an interplay of dietary, genetic and environmental factors [3,5]. Various studies have shown the prevalence of PMGH to range from 28% to almost 59% [6,7,8,9]. Hormonal changes, career choices, family history, stress, hair care, personal hygiene, serum ferritin, and Vitamin D3 have been listed as risk factors for PMGH in various studies, and these factors in turn are thought to affect the ability to regenerate hair pigment [9,10,11,12,13,14,15,16,17]. MBBS students were noted to have a higher prevalence of PMGH (43% to 59%), and stress could probably be an underlying cause in this scenario [9]. However, there are very limited studies of PMGH in certain geographical areas like the Middle East region. Keeping these factors in mind, the present study was conducted among MBBS students in Saudi Arabia. The objective of this study was to estimate the prevalence of premature canities among university students and to establish correlates for premature canities based on associated risk factors. The secondary objectives were to assess their habits regarding hair care and their attitudes and beliefs regarding PMGH.

## 2. Methodology

This study was a cross-sectional observational study, employing a convenience (non-probability) sampling technique for participant recruitment. This study was conducted at a medical college in the eastern province of Saudi Arabia during the academic year 2023–2024. The Institutional Review Board approval was sought prior to initiating the study, (Ref. No. KFU-REC-2023-DEC-ETHICS1768). Participants who were unwilling, or who had premature graying or vitiligo were excluded from this study.

Data collection was carried out through an online, self-administered questionnaire distributed via Google form, and the link was shared across social media platforms such as Facebook, WhatsApp, Telegram, Email, and Twitter. The goals of this study were clearly explained in the online form. The survey included consent, and no identifying information was sought. A validated questionnaire, derived from previous studies, was employed to ensure reliability. It included sociodemographic factors, possible risk factors for premature graying of hair (PMGH) and its effects. A small pilot study involving 239 participants was carried out.

Data was analyzed using the Statistical Package for the Social Sciences (SPSS), version 26 (Released 2019, IBM Corp., Armonk, NY, USA). All statistical tests were two-tailed, and the level of significance was set at *p* ≤ 0.05. Descriptive statistics were performed for categorical variables using frequencies and percentages, while numerical data were presented as means with standard deviations. Various factors including PMGH data, behavioral patterns, use of hair products, family history, and attitudes were organized into tables for analysis. For data presentation, Microsoft Excel was used to generate graphical representations. The relationships between categorical variables were examined using the Pearson chi-square test to assess statistical significance, and exact probability tests were applied when the data involved small frequency distributions.

## 3. Results

A total of 239 eligible students were included, with ages ranging from 18 to 30 years and a mean age of 22.9 ± 5.3 years. The study sample consisted of 130 (54.4%) females and 109 (45.6%) males (Table 1). 89 students (37.2%; 95% CI: 31.1% to 43.4%) had PMGH. Of these, 46% of patients reported onset of PMGH in the age group 19–22 years and, 2.2% of participants had an onset before 10 years. Scalp was noted to be the commonest site of PMGH (94.4%), and, in the scalp, the frontal area was most affected (33.7%) (Table 2). Almost 55% of candidates reported mild PMGH (<10 gray hairs), and 62% reported slow progression (<3 gray hairs/month). No statistically significant association was noted between PMGH and past medical or dermatological illnesses (Table 3). Family history of premature graying was statistically significant (*p* = 0.001%) (Table 4). No significant relationship was noted between use of supplements, hair oils or Minoxidil use (Table 5). Regarding habits and diet, smoking, keto diet and previous history of hair dye use were found to be associated with PMGH with a significant *p*-value < 0.05 (Table 6). Regarding students’ attitude to PMGH, the majority of students felt that hair washing, oiling and moisturization have no relation to PMGH (Table 7). Family history of PMGH was found to be a significant association (*p* = 0.01) (Table 8).

## 4. Discussion

**Figure 1:**
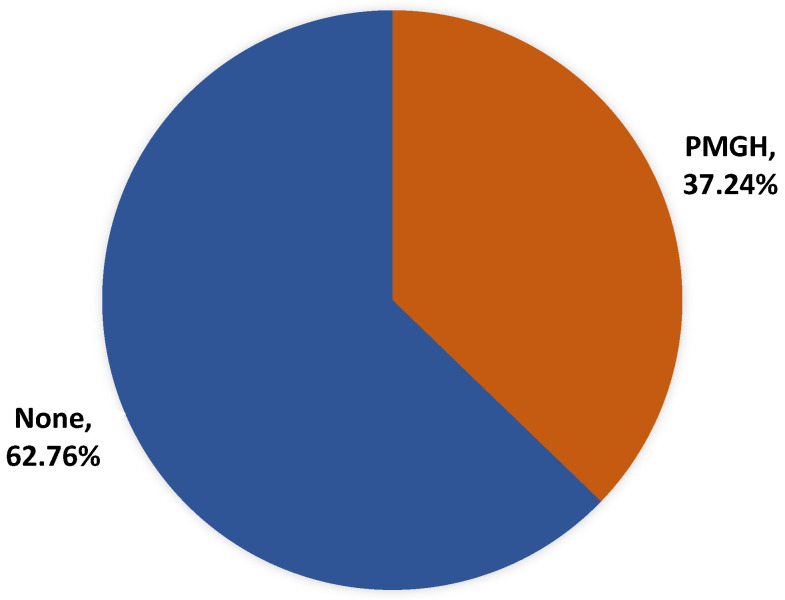
Prevalence of premature graying of hair (n = 239).

In total, 239 medical students participated in an online cross-sectional study conducted at a medical college in Saudi Arabia. All the students were in the 18–30 year age group, with the majority in the 19–24 year age group. There were 109 males and 130 females. In total, 37.24% of students had premature graying Figure 1. A study conducted at a Saudi University found a prevalence of PMGH of 42.5% [6]. A Nepali Medical College survey found a prevalence of 40% and studies conducted among college students found prevalence ranging from 27.3% to 34.5% [8,11,12,17].

According to the results of our study on grading severity, 55.1% of students had mild early canities, 30.3% had moderate canities, and 5.6% had severe canities. The commonest area affected by PMGH was found to be the scalp and especially the frontal area, whereas a Turkish study noted it more in the parietal and temporal area [12].

Among the subjects with PMGH, positive family history was noted to be a significant association. This was similar to studies elsewhere [8,10,11,12,17,18,19]. Smoking was found to be a statistically significant association in the present study (*p* value = 0.03%), as in other studies in this age group [6,8,11], whereas one study found no significant relation between the two [10]. Intake of keto diet was found to be a significant risk factor in this study [*p* values 0.10, 0.04]. Other studies reported iron deficiency as a significant association [8]. Students who had a history of previously changing hair color were found to have a significant association with PMGH (*p* = 0.042). A study conducted in Saudi Arabia noted hair straightening, dryer and gel use to be associated. Students in this study felt that cleaning, oiling and moisturizing hair had no relation to premature graying.

To date, there are no studies that specifically address premature graying of hair among medical students in this area in the Kingdom of Saudi Arabia. Our study found that 37.24% of medical students had premature graying. Risk factors in our study with significant association were a combination of genetic and environmental factors like family history, smoking, keto diet, and coloring of hair. Although the genetic part is unmodifiable, risk factors like smoking could be terminated. Students and patients should be advised regarding the modifiable risk factors. The importance of a healthy lifestyle is again highlighted by the results of this study.

Premature Hair Graying (PMGH) poses both cosmetic and psychological concerns for the affected individuals. While it is difficult to prevent premature graying, a variety of treatment options aims at addressing the root cause and restoring hair color. In order to focus on potential remedies, numerous attempts have been made in the literature to comprehend the fundamental mechanism of PMGH. Nonetheless, no studies have yet described a long-term method of reversing it. Factors reducing oxidative stress and speeding up melanocyte growth are the focus of future treatment. Additional research on these modalities of treatment is required.

## 5. Conclusions

PMGH is a condition that affects a significant portion of the population and can have a profound impact on the physical and psychological well-being of individuals. The results of this study demonstrate that early hair graying is caused by both genetic and modifiable factors, and its incidence and psychosocial effects might be lessened with increased awareness and early lifestyle changes. The findings of this study could have a positive impact on students who are affected by this condition and provide an improved understanding of factors influencing hair loss. Health education programs emphasizing smoking cessation, balanced nutrition, and cautious use of cosmetic hair products could help reduce the risk and early onset of PMGH among young adults. Additionally, promoting awareness about the importance of adequate vitamin and mineral intake (e.g., vitamins B12, D, iron, and copper) may support preventive efforts. In order to determine the specific etiologic significance of these variables and develop a range of prevention and treatment targets for PHG, further studies with a larger sample size are needed.

### Strengths and Weaknesses

This study demonstrates several notable strengths. In the first place, it fills a significant local data gap by being one of the few studies looking at early graying of hair among medical students in the eastern province of Saudi Arabia. Furthermore, this study’s sample size of 239 participants was thought to be sufficient, giving it the statistical power to identify any possible associations. The instrument’s clarity and internal validity were improved by using a previously validated questionnaire that had also undergone pilot testing. Furthermore, this study’s comprehensive inclusion of a wide range of sociological, lifestyle, medical, and family history variables allowed for the identification of multiple and diverse correlations with premature graying of hair (PMGH).

However, this study also has several weaknesses. The cross-sectional design limits the ability to establish causal relationships between PMGH and potential risk factors such as smoking or dietary habits; it only allows for the identification of associations. The use of convenience sampling through online forms and social media may have introduced selection bias, thereby restricting the generalizability of the findings. Moreover, the reliance on self-reported data regarding the onset and severity of PMGH, as well as lifestyle behaviors, increases the likelihood of recall and reporting biases. This study’s external validity is also limited because the sample was mostly medical students, which makes it hard to apply the results to other groups of students or to young adults in general. Lastly, the absence of biochemical measurements weakens this study’s capacity to confirm laboratory-based deficiencies, as key factors such as vitamin D, iron, and vitamin B12 levels were assessed solely through self-reporting.

## Figures and Tables

**Table 1:** Personal and academic data of study students at King Faisal University in Al Ahsa, Saudi Arabia (n = 239).

Personal Data	N	%
**Age in years**		
≤18	2	0.8%
19–24	212	88.7%
25–30	25	10.5%
**Gender**		
Male	109	45.6%
Female	130	54.4%
**Marital status**		
Single	213	89.1%
Married	26	10.9%

**Table 2:** Premature graying of hair-related data among study students (n = 89).

PMGH	No.	%
**Age of onset (years) when your hair began to gray prematurely**		
<10 years old	2	2.2%
11–14 years old	11	12.4%
15–18 years old	25	28.1%
19–22 years old	41	46.1%
23–26 years old	9	10.1%
27–29 years old	1	1.1%
**Where do you see the white hair more?**		
Scalp hair	84	94.4%
Beard and mustache hair	2	2.2%
Eyebrows	2	2.2%
Eyelashes	1	1.1%
**Which of the following areas of the head hair do you notice graying of your hair at presentation?**		
Frontal area	30	33.7%
Vertex area	19	21.3%
Parietal	18	20.2%
Diffuse graying at presentation	12	13.5%
Temporal area	7	7.9%
Occiput area	2	2.2%
Nuchal area	1	1.1%
**Progression rate**		
Rapid (≥3 total gray hairs/month)	12	13.5%
Slow (<3 total gray hairs/month)	55	61.8%
I don’t know	22	24.7%
**Severity grading**		
Mild (<10 total gray hairs)	49	55.1%
Moderate (10–100 total gray hairs)	27	30.3%
Severe (>100 total gray hairs)	5	5.6%
I don’t know	8	9.0%
Gender		
Male	40	36.7%
Female	49	37.7%
Marital status		
Single	76	35.7%
Married	13	50.0%

**Table 3:** Medical and dermatological history in PMGH.

Medical History	Total		PMGH				*p*-Value
			Yes		No		
	N	%	N	%	N	%	
**Do you have any history of chronic disease?**							0.571
Yes	26	10.9%	11	42.3%	15	57.7%	
No	213	89.1%	78	36.6%	135	63.4%	
**Do you suffer from any previous skin disorder?**							0.257
Yes	75	31.4%	24	32.0%	51	68.0%	
No	164	68.6%	65	39.6%	99	60.4%	
**If yes, mention**							0.698 ^
Acne Vulgaris	23	30.7%	6	26.1%	17	73.9%	
Atopic dermatitis	2	2.7%	1	50.0%	1	50.0%	
Dandruff	2	2.6%	1	50.0%	1	50.0%	
Eczema	22	29.3%	9	40.9%	13	59.1%	
Folliculitis	1	1.3%	0	0.0%	1	100.0%	
I don’t know	7	9.3%	3	42.9%	4	57.1%	
juvenile dermatomyositis	1	1.3%	1	100.0%	0	0.0%	
Pitryasis versicolos	1	1.3%	0	0.0%	1	100.0%	
Psoriasis	5	6.7%	1	20.0%	4	80.0%	
Rash on my back and warts	1	1.3%	1	100.0%	0	0.0%	
Rosacea/seborrheic dermatitis	1	1.3%	0	0.0%	1	100.0%	
Seborrheic dermatitis	1	1.3%	0	0.0%	1	100.0%	
Sun allergy	1	1.3%	0	0.0%	1	100.0%	
Tenia capitis	1	1.3%	0	0.0%	1	100.0%	
Urticaria	5	6.7%	1	20.0%	4	80.0%	
Viral infection of the skin	1	1.3%	0	0.0%	1	100.0%	
**Atopic dermatitis**							0.879
Yes	36	15.1%	13	36.1%	23	63.9%	
No	203	84.9%	76	37.4%	127	62.6%	
**Hair loss**							0.342
Yes	141	59.0%	56	39.7%	85	60.3%	
No	98	41.0%	33	33.7%	65	66.3%	
**Dandruff**							0.278
Yes	129	54.0%	44	34.1%	85	65.9%	
No	110	46.0%	45	40.9%	65	59.1%	
**Did you test positive for COVID-19 virus?**							0.958
Yes	116	48.5%	43	37.1%	73	62.9%	
No	123	51.5%	46	37.4%	77	62.6%	

*p*: Pearson X^2^ test. ^: Exact probability test.

**Table 4:** Family history in PMGH.

Family History	Total		PMGH				*p*-Value
			Yes		No		
	N	%	N	%	N	%	
**Does anyone in your family have premature grey hair?**							0.001 *
Yes	149	62.3%	71	47.7%	78	52.3%	
No	90	37.7%	18	20.0%	72	80.0%	

*p*: Pearson X^2^ test. * *p* < 0.05 (significant).

**Table 5:** Hair care habits of students.

	Total		PMGH				*p*-Value
			Yes		No		
	N	%	N	%	N	%	
**Zinc supplementation**							0.863
Yes	39	16.3%	15	38.5%	24	61.5%	
No	200	83.7%	74	37.0%	126	63.0%	
**Iron supplementation**							0.812
Yes	89	37.2%	34	38.2%	55	61.8%	
No	150	62.8%	55	36.7%	95	63.3%	
**Vitamin D supplementation**							0.836
Yes	139	58.2%	51	36.7%	88	63.3%	
No	100	41.8%	38	38.0%	62	62.0%	
**Vitamin B12 supplementation**							0.483
Yes	61	25.5%	25	41.0%	36	59.0%	
No	178	74.5%	64	36.0%	114	64.0%	
**Chemotherapeutic drugs**							0.466 ^
Yes	8	3.3%	2	25.0%	6	75.0%	
No	231	96.7%	87	37.7%	144	62.3%	
**Use of minoxidil as treatment of hair fall**							0.332
Yes	36	15.1%	16	44.4%	20	55.6%	
No	203	84.9%	73	36.0%	130	64.0%	
**If you answered “yes”, did you notice increased number of white hairs after using the minoxidil?**							-
Yes	10	32.3%	10	32.3%	-	-	
No	21	67.7%	21	67.7%	-	-	
**Use of rosemary oil**							0.532
Yes	72	30.1%	29	40.3%	43	59.7%	
No	167	69.9%	60	35.9%	107	64.1%	
**If you used rosemary oil did you notice increased number of white hair?**							
Yes	14	29.2%	14	29.2%	-	-	
No	34	70.8%	34	70.8%	-	-	

*p*: Pearson X^2^ test. ^: Exact probability test.

**Table 6:** Dietary and personal habits of students related to hair.

Habits/Diet	Total		PMGH				*p*-Value
			Yes		No		
	N	%	N	%	N	%	
**Smoking**							0.003 *^
Yes	10	4.2%	8	80.0%	2	20.0%	
No	227	95.0%	79	34.8%	148	65.2%	
Ex-smoker	2	0.8%	2	100.0%	0	0.0%	
**What is your diet?**							0.871 ^
Mixed	202	84.5%	74	36.6%	128	63.4%	
Mostly meat	34	14.2%	14	41.2%	20	58.8%	
Vegetarian	3	1.3%	1	33.3%	2	66.7%	
**Did you follow keto diet?**							0.013 *^
Yes	11	4.6%	8	72.7%	3	27.3%	
No	228	95.4%	81	35.5%	147	64.5%	
**If you follow keto diet did you notice increased number of white hairs**							-
Yes	7	28.0%	7	28.0%	0	0.0%	
No	18	72.0%	18	72.0%	0	0.0%	
**Do you have regular exposure to sunlight?**							0.676
Yes	74	31.0%	29	39.2%	45	60.8%	
No	165	69.0%	60	36.4%	105	63.6%	
**Duration of sun exposure**							0.103 ^
<1 h	28	37.8%	15	53.6%	13	46.4%	
<2 h	25	33.8%	9	36.0%	16	64.0%	
2 h	5	6.8%	0	0.0%	5	100.0%	
3/more h	16	21.6%	5	31.3%	11	68.8%	
**Have you changed the color of your hair previously?**							0.042 *
Yes	41	17.2%	21	51.2%	20	48.8%	
No	198	82.8%	68	34.3%	130	65.7%	
**Do you use henna-based natural color?**							0.813
Yes	72	30.1%	26	36.1%	46	63.9%	
No	167	69.9%	63	37.7%	104	62.3%	
**Do you use hair straightener?**							0.322
Yes	95	39.7%	39	41.1%	56	58.9%	
No	144	60.3%	50	34.7%	94	65.3%	
**Do you use any hair dryer?**							0.867
Yes	87	36.4%	33	37.9%	54	62.1%	
No	152	63.6%	56	36.8%	96	63.2%	
**Do you use any hair gel?**							0.900
Yes	58	24.3%	22	37.9%	36	62.1%	
No	181	75.7%	67	37.0%	114	63.0%	
**Do you use any hair conditioner?**							0.753
Yes	115	48.1%	44	38.3%	71	61.7%	
No	124	51.9%	45	36.3%	79	63.7%	

*p*: Pearson X^2^ test. ^: Exact probability test. * *p* < 0.05 (significant).

**Table 7:** Study of students’ attitude and perception towards PMGH by their disease experience.

Attitude	Total		PMGH			
			Yes		No	
	No	%	No	%	No	%
**Do you think that not washing your hair and taking care of its cleanliness has anything to do with the appearance of premature white hair?**						
Yes	49	20.5%	19	21.3%	30	61.2%
No	190	79.5%	70	78.7%	120	63.2%
**Do you think that not using oil and moisturizing the hair is one of the reasons for the appearance of premature white hair?**						
Yes	49	20.5%	20	22.5%	29	59.2%
No	190	79.5%	69	77.5%	121	63.7%

**Table 8:** Family history and PMGH.

Family History	Total		PMGH				*p*-Value
			Yes		No		
	N	%	N	%	N	%	
**Does anyone in your family have premature grey hair?**							0.001 *
Yes	149	62.3%	71	47.7%	78	52.3%	
No	90	37.7%	18	20.0%	72	80.0%	
**Who is the person in your family who suffers from premature graying of the hair?**							
Mother	72	30.1%	36	50.0%	36	50.0%	
Father	68	28.5%	34	50.0%	34	50.0%	
Sister	64	26.8%	36	56.3%	28	43.8%	
Brother	54	22.6%	31	57.4%	23	42.6%	
Aunt	39	16.3%	17	43.6%	22	56.4%	
Uncle	38	15.9%	17	44.7%	21	55.3%	
Grandparent	35	14.6%	15	42.9%	20	57.1%	
I don’t know	90	37.7%	21	23.3%	69	76.7%	
**Do they have any history of chronic disease?**							0.166
Yes	50	20.9%	20	40.0%	30	60.0%	
No	118	49.4%	49	41.5%	69	58.5%	
I do not know	71	29.7%	20	28.2%	51	71.8%	
**Do they suffer from any previous skin disorder?**							0.043 *
Yes	40	16.7%	18	45.0%	22	55.0%	
No	122	51.0%	51	41.8%	71	58.2%	
I do not know	77	32.2%	20	26.0%	57	74.0%	
**Do they suffer from any previous hair problems?**							0.017 *
Yes	42	17.6%	20	47.6%	22	52.4%	
No	108	45.2%	46	42.6%	62	57.4%	
I do not know	89	37.2%	23	25.8%	66	74.2%	

*p*: Pearson X^2^ test. * *p* < 0.05 (significant).

## Data Availability

The data supporting the findings of this study are available from the corresponding author upon reasonable request, subject to ethical and privacy restrictions.

## Abbreviations

PMGH	Premature Graying of Hair
MBBS	Bachelor of Medicine, Bachelor of Surgery

## References

[1] Kocaman S.A., Çetin M., Durakoğlugil M.E., Çanga A., Çiçek Y., Doğan S., Şahin İ., Şatıroğlu Ö. (2012). The degree of premature hair graying as an independent risk marker for coronary artery disease: A predictor of biological age rather than chronological age. Anatol. J. Cardiol..

[2] Anggraini D.R., Feriyawati L., Hidayat H., Wahyuni A.S. (2019). Risk factors associated with premature hair greying of young adult. Open Access Maced. J. Med. Sci..

[3] Kumar A.B., Shamim H., Nagaraju U. (2018). Premature graying of hair: Review with updates. Int. J. Trichol..

[4] Das S., Chander R., Garg T., Mendiratta V., Singh R., Sanke S. (2023). Cardiovascular risk markers in premature canities. Indian J. Dermatol. Venereol. Leprol..

[5] Daulatabad D., Singal A., Grover C., Chhillar N. (2016). Profile of Indian patients with premature canities. Indian J. Dermatol. Venereol. Leprol..

[6] Almutairi R.T., Al Dhafiri M. (2019). Premature greying of hair among the population of King Faisal University in Al-Ahasa, Saudi Arabia: An epidemiological study. Int. J. Med. Dev. Ctries..

[7] Alkahtani A., Alfadhli R., Aldakhilallah M., Almutlaq M., Alzamil F. (2022). Relationship between premature hair graying and psychological stress: A cross-sectional study on young Saudis in Riyadh, Saudi Arabia. Int. J. Med. Dev. Ctries..

[8] Kansal S., Bilimale A.S., Gopi A., Bv S. (2021). Premature Hair Greying—Magnitude and Associated Factors: A cross-sectional study in a university in Mysuru. Indian J. Community Health.

[9] Singh R., Madke B., Bansod S., Yadav N. (2021). Premature graying of hair: A concise review. Cosmoderma.

[10] Acer E., Arslantaş D., Emiral G.Ö., Ünsal A., Atalay B.I., Göktaş S. (2020). Clinical and epidemiological characteristics and associated factors of hair graying: A population-based, cross-sectional study in Turkey. An. Bras. Dermatol..

[11] Bhola N., Valendu G., Ranjeeta K. (2020). A Community Based Study to Estimate Prevalence and Determine Correlates of Premature Graying of Hair among Young Adults in Srinagar, Uttarakhand, India. Int. J. Trichol..

[12] Saad M., Babar N.F., Majeed R., Rehman A.U., Khan O.A., Chatha D.E., Aamir U., Nadeem A., Abbas S. (2019). Impact of Premature Greying of Hair on Socio-cultural Adjustment and Self-esteem among Medical Undergraduates in Foundation University, Islamabad. Cureus.

[13] Shin H., Ryu H.H., Yoon J., Jo S., Jang S., Choi M., Kwon O., Jo S.J. (2015). Association of premature hair graying with family history, smoking, and obesity: A cross-sectional study. J. Am. Acad. Dermatol..

[14] Zayed A.A., Shahait A.D., Ayoub M.N., Yousef A.M. (2013). Smokers’ hair: Does smoking cause premature hair graying?. Indian Dermatol. Online J..

[15] Bhat R.M., Sharma R., Pinto A.C., Dandekeri S., Martis J. (2013). Epidemiological and Investigative Study of Premature Graying of Hair in Higher Secondary and Pre-University School Children. Int. J. Trichol..

[16] El-Husseiny R., Alrgig N.T., Fattah N.S.A. (2021). Epidemiological and biochemical factors (serum ferritin and vitamin D) associated with premature hair graying in Egyptian population. J. Cosmet. Dermatol..

[17] Chaudhary S., Mahotra N.B. (2023). Early Canities among Undergraduate Medical Students of a Medical College: A Descriptive Cross-sectional Study. J. Nepal. Med. Assoc..

[18] Padmavathi P., Sathiyapriya V., Jos P., Sudha V. (2022). Prevalence of premature canities among college students studying in a private medical college, Chennai: A cross sectional study. Int. J. Health Sci..

[19] Lubis E.Z.S., Jusuf N.K. (2020). Risk Factors Analysis of Premature Canities in Medical Students of Universitas Sumatera Utara Class 2016–2018. Sumat. Med. J..

